# Novel imaging and biophysical approaches to study tissue hydraulics in mammalian folliculogenesis

**DOI:** 10.1007/s12551-024-01231-4

**Published:** 2024-10-07

**Authors:** Jake Turley, Kim Whye Leong, Chii Jou Chan

**Affiliations:** 1 Mechanobiology Institute, National University of Singapore, Singapore, Singapore; 2 Department of Biological Sciences, National University of Singapore, Singapore, Singapore

**Keywords:** Ovary, Folliculogenesis, Ovulation, Biophotonics, Machine learning, Tissue mechanics

## Abstract

A key developmental stage in mammalian folliculogenesis is the formation of a fluid-filled lumen (antrum) prior to ovulation. While it has long been speculated that the follicular fluid is essential for oocyte maturation and ovulation, little is known about the morphogenesis and the mechanisms driving the antrum formation and ovulation, potentially due to challenges in imaging tissue dynamics in large tissues. Misregulation of such processes leads to anovulation, a hallmark of infertility in ageing and diseases such as the polycystic ovary syndrome (PCOS). In this review, we discuss recent advances in deep tissue imaging techniques, machine learning and theoretical approaches that have been applied to study development and diseases. We propose that an integrative approach combining these techniques is essential for understanding the physics of hydraulics in follicle development and ovarian functions.

## Introduction

**Fig. 1:**
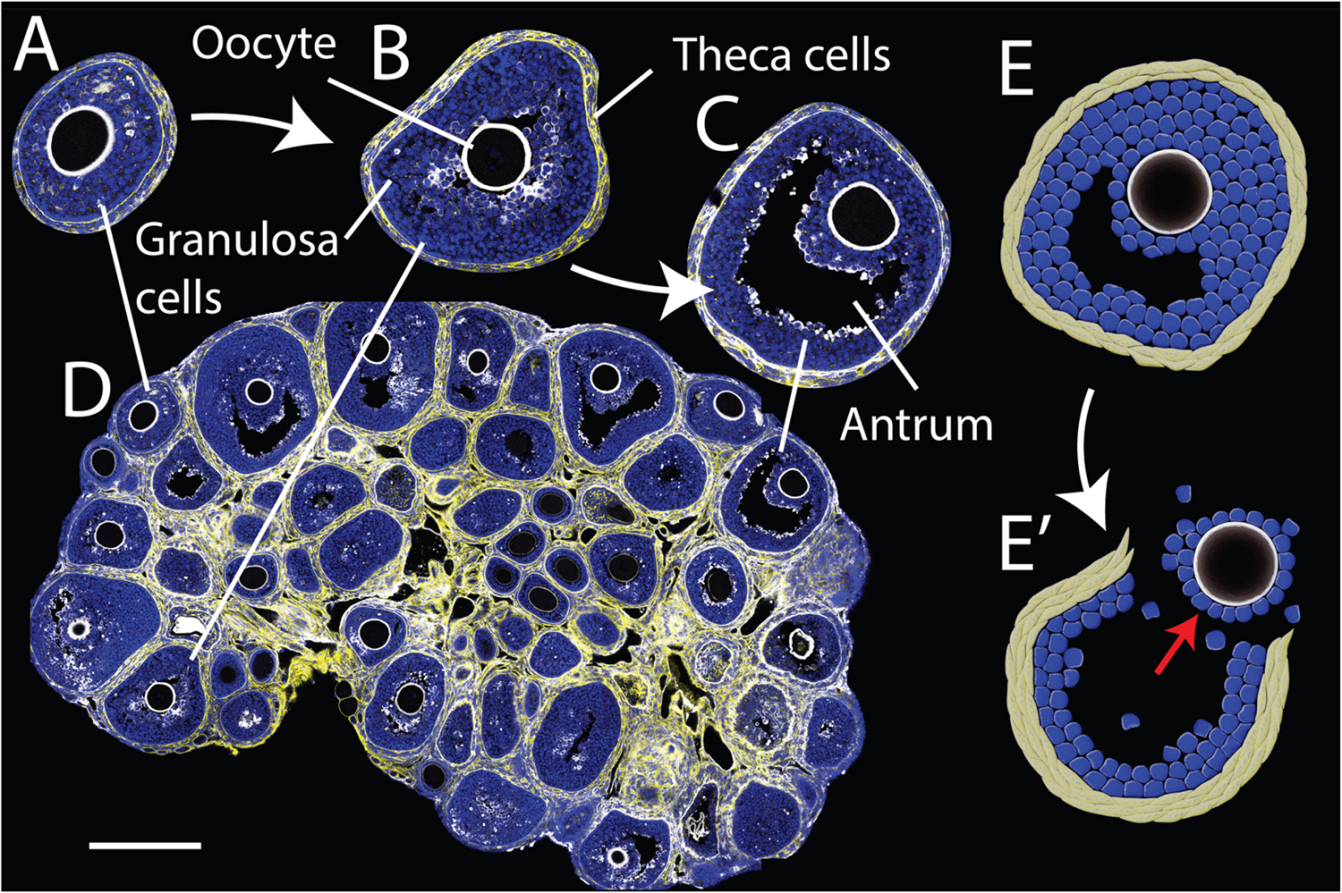
Hydraulic control of late-stage ovarian follicle development. **A** Early secondary follicle (100–180 $\mu$m). **B** Secondary follicle with small pockets of fluids (180–300 $\mu$m). **C** Pre-ovulatory follicle with fully formed “smiley-faced” antrum (300–400 $\mu$m). **D** A mouse ovarian tissue slice with DNA (Blue), pan-collagen (yellow) and anti-Müllerian hormone (grey). Scale bar, 300 $\mu$m. **E-E’** Ovulation involves the rupture of the follicle wall and the release of oocyte, though the exact mechanism remains poorly understood

The development of functional oocytes followed by successful ovulation is a critical process in early mammalian reproduction (Biswas et al. 2022; Telfer et al. 2023). During folliculogenesis, the follicle morphology provides a rough classification of its stage of development (Fig. 1). In the secondary follicle stage, the follicle consists of an oocyte surrounded by multi-layered granulosa cells (GC) with an outer layer of elongated theca cells (TC) (Fig. 1B) (Rodgers and Irving-Rodgers 2010; Biswas et al. 2022). The formation of a fluid-filled lumen, also known as the antrum, begins as small pockets of interstitial fluids which nucleate between the GCs. As the fluid volume increases, these fluids evolve to form a singly resolved antrum showing a stereotypical “smiley” pattern in the case of mouse ovaries (Fig. 1C) (Rodgers and Irving-Rodgers 2010; Biswas et al. 2022). However, the exact mechanisms for the *de novo* luminogenesis during antrum development remain largely unknown. As the follicles grow further, and with the stimulation of luteinizing hormones, they rupture and release the fluid, along with the ejection of the cumulus-oocyte complex (Fig. 1D) (Converse et al. 2023; Zaniker et al. 2023; Komatsu and Masubuchi 2017; Matsuzaki 2021).

While extensive studies in the past have identified various key signalling pathways controlling oocyte growth, less is known about the roles of mechanical stress and biophysics in folliculogenesis (Biswas et al. 2022; Prasasya and Mayo 2019; Telfer et al. 2023). In recent years, tissue mechanics has emerged as a central regulator of various developmental processes, such as tissue folding and patterning (Matsuzaki 2021; Bevilacqua et al. 2023; Zhang and Fodor 2023; Biswas et al. 2022, 2024; Athilingam et al. 2021; Barriga et al. 2018; Fiorentino et al. 2023). In mice, mechanical stress exerted by the extracellular matrix (ECM) or the TCs surrounding the follicles have been shown to influence follicle activation and growth (Nagamatsu et al. 2019; Biswas et al. 2024). During ageing, the aged ovaries have shown significant increase in the ECM stiffness (Mara et al. 2020; Fiorentino et al. 2023), which correlates with impaired follicle and oocyte functions (Pietroforte et al. 2024) and ovulation (Umehara et al. 2022). In PCOS patients, the ovaries are characterised by multiple cystic-like follicles that fail to ovulate (Mara et al. 2020; Lee et al. 2024c; Fiorentino et al. 2023), suggesting that impaired luminogenesis and follicle rupture may contribute to developmental arrest in ovarian disease and ageing.

Indeed, in addition to cell-generated cytoskeleton forces, there has been increasing evidence that tissue-scale fluid forces can transmit long-range mechanical signals to influence morphogenesis and cellular functions during development (Chan and Hirashima 2022; Chan and Hiiragi 2020). However, how tissue hydraulics impacts mammalian follicle development has not been addressed so far. One reason for this is the challenge of studying tissue dynamics in large organs. In this review, we highlight recent advances in deep tissue imaging techniques, machine learning and theoretical models that have been increasingly applied to study complex 3D tissue dynamics in vivo. We discuss how these approaches, when applied individually or together, could deepen our basic understanding of late-stage folliculogenesis (for mechanobiology of earlier stages, see recent review (Telfer et al. 2023; Prasasya and Mayo 2019; Biswas et al. 2022)), with important clinical implications in future infertility studies and assisted reproductive technology.

## Non-invasive and label-free imaging techniques to study tissue dynamics during folliculogenesis

In the study of late-stage ovarian folliculogenesis, the use of intravital imaging combined with histological analysis of fixed ovary tissues has helped to establish a general understanding of the developmental processes (Feng et al. 2018). However, the tissue dynamics of these morphological events are less well understood. To do so, direct live imaging of the spatiotemporal dynamics of cellular and fluid movements is required. Here, we discuss recent advances in the development of novel biophotonic tools that have the potential to overcome current imaging limitations and provide new mechanistic insights to our understanding of fluid antrum formation and ovulation.

### Current imaging techniques utilised in ovarian studies

In the past, measurements of fluid fraction using ultrasonography have shed light on lumen expansion during follicle growth (Rodgers and Irving-Rodgers 2010). Alongside the identification of the ECM at the GC apical surface in electron micrographs, it was hypothesised that the directional secretion of large molecules, such as proteoglycans and hyaluronans, establishes an osmotic gradient against the surrounding thecal vasculature, thereby recruiting fluid for lumen growth (Clarke et al. 2006). In the study of ovulation, intravital imaging of blood vessel thickness and blood flow surrounding the ovary using multiphoton microscopy (Migone et al. 2013) and ultrasonography helped reveal the mechanism of endothelin-induced contractions of smooth muscle cells, causing basolateral invaginations prior to ovulation (Migone et al. 2016). Subsequent work using serial tissue section indirectly suggested complementary action from the inward migration of mural GCs following the surge of luteinizing hormones (Owen and Jaffe 2023). Nevertheless, ultrasonography-based intravital imaging is constrained by its poor spatial resolution of a few millimeters. In contrast, multiphoton microscopy offers better spatial resolution but is limited to a depth penetration of about 200 $\mu$m in ovarian tissues (Migone et al. 2016) and has significantly slower imaging speeds. Additionally, multiphoton microscopy requires the use of fluorescent labels, which may incur phototoxic effects. While transgenic animal lines expressing fluorescent proteins are useful, such an approach is typically restricted to mice, making it difficult to translate to other mammalian species and humans. Another imaging approach is ultrastructural studies using electron microscopy. While these studies have led to the discovery of novel features such as the Call-Exner bodies during luminogenesis (Gosden et al. 1989; Van Wezel et al. 1999), they remain largely descriptive and do not inform the dynamic processes underlying luminogenesis. To address this, other label-free imaging techniques may be required, as outlined below.

### Optical coherence tomography

Here, we introduce optical coherence tomography (OCT), an emerging label-free modality with intermediate depth penetration and resolution used in intravital imaging. OCT works on the basis of detecting back-scattered signals, analogous to ultrasound, except that OCT uses light rather than acoustic waves which therefore offers higher spatial resolutions of 1–15 $\mu$m (Fujimoto 2003; Chow et al. 2024), with reasonable penetration depths of up to 1 mm during intravital imaging of ovarian tissues. As a label-free technique, OCT is not bound by a “fluorescence budget” as is the case for confocal microscopy, allowing for prolonged imaging without signal degradation. Typically used in ophthalmic diagnosis and research (Everett et al. 2021), OCT has recently been applied to reproductive biology (Burton et al. 2015) as an optical biopsy tool for assessing ovarian reserve and detecting metastases (Takae et al. 2017, 2018; Peters et al. 2016). Owing to the high optical contrast between different tissue components and its high-speed volumetric imaging capability, OCT has been employed to capture real-time dynamics of oocytes and pre-implantation embryo transport in the mouse oviduct, revealing uncharted location-dependent movement trajectories (Wang and Larina 2021; Umezu and Larina 2023).

**Fig. 2:**
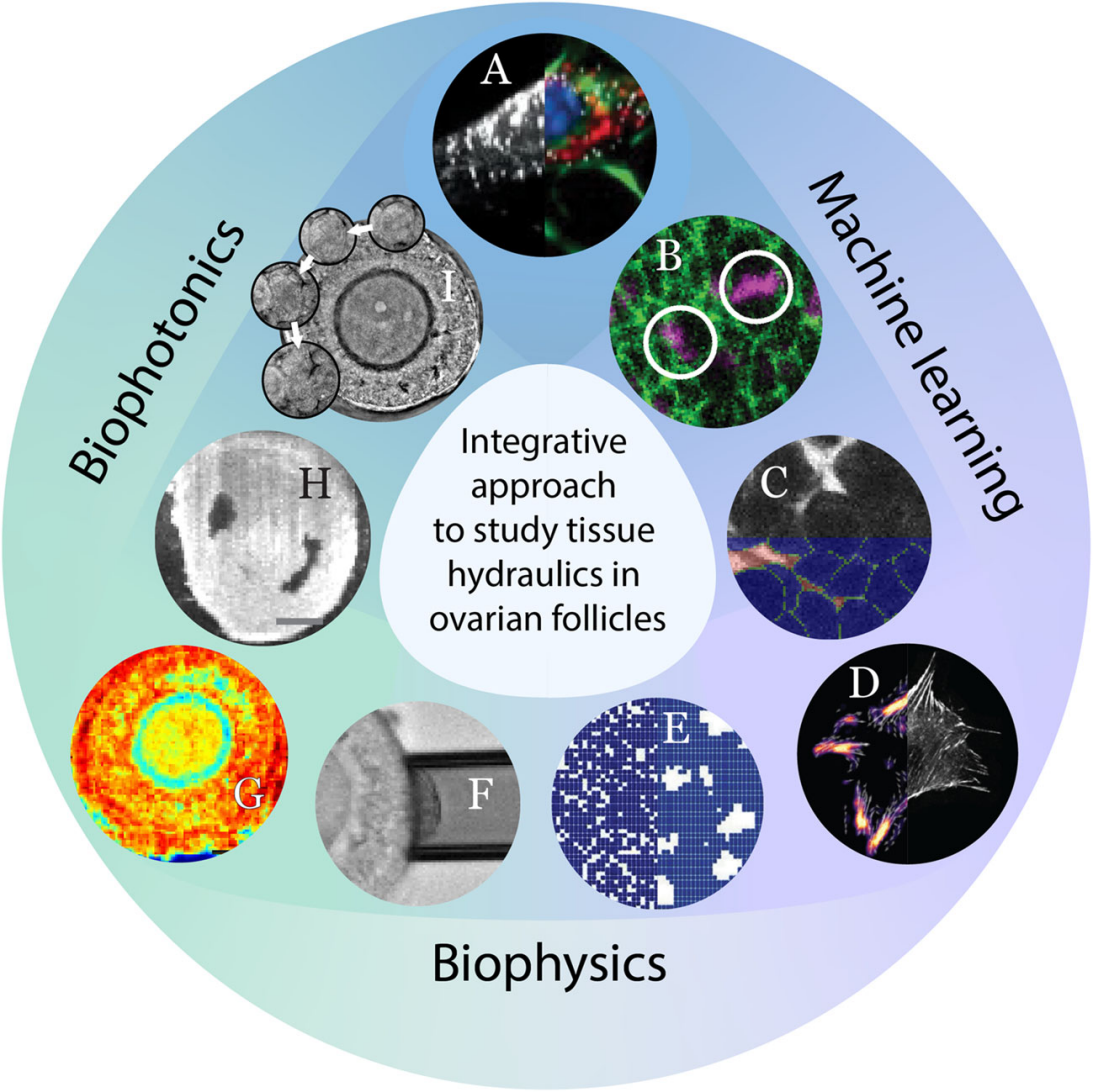
Investigating tissue hydraulics in mammalian ovarian folliculogenesis using an integrative approach that combines advanced biophotonics, deep learning and biophysical modelling. **A** Label-free RI imaging combined with deep learning enables visualisation of subcellular structures and dynamics with high specificity (Jo et al. 2021). **B** Deep learning model detects cell divisions during wound healing with high accuracy (Turley et al. 2024a). **C** 3D segmentation of GCs in the ovarian follicle. **D** Machine learning based on physics-informed neural networks allows inference of traction forces in a cell (Schmitt et al. 2024). **E** Computational approach to model luminogenesis in follicles based on fluid mixing-demixing transition. **F** Measurement of the surface tension of ovarian follicle using micropipette aspiration. **G** Representative “stiffness map” of an ovarian follicle imaged by Brillouin microscopy, revealing clear intrafollicular mechanical heterogeneities (Chan et al. 2021). **H** An image of pre-ovulatory follicle acquired with OCM. Scale bar, 100 $\mu$m. **I** Images showing a cell undergoing division, acquired through QPI. Interstitial fluids are also visible due to its distinct RI from cellular bodies

Nevertheless, intravital imaging using OCT requires precise orientation of the imaged organs, and can be exacerbated by tissue motion such as muscle contraction and breathing (Burton et al. 2015). To overcome these limitations in imaging ovaries, advancement in ex vivo cultures such as 3D follicle culture (Converse et al. 2023), multifollicle tissue (Biswas et al. 2022) and slice culture of ovaries (Komatsu et al. 2018) can be utilised. Furthermore, ex vivo culture is more amenable to optical coherence microscopy (OCM), a variant of OCT that offers higher spatial resolution with reduced imaging depth (Aguirre et al. 2015). This has been demonstrated in live imaging of rapid fluid dynamics and muscle hydraulics in sea animals such as cnidarian *Nematostella vectensis* (Stokkermans et al. 2022) and freshwater sponge *Spongilla lacustris* (Ruperti et al. 2024). In a similar vein, we propose that OCM will be a powerful tool for studying antrum formation and ovulation in mammalian species, allowing for unprecedented tracking of lumen fluid growth and organisation and cellular dynamics during follicle rupture in ex vivo culture (Fig. 2H). Recently, other applications of OCM in vivo have also been explored, expanding its potential for future use in intravital imaging (Moore et al. 2019) and imaging-based biophysical measurements (see the “Biophysical control of ovulation” section).

### Quantitative phase imaging

Building on existing phase measurement techniques in OCT (Nguyen et al. 2022; Park et al. 2018), quantitative phase imaging (QPI) technique was developed as another fast, label-free and non-invasive technique for biological imaging. A detailed review of the underlying principles and the different applications in biomedicine has been published (Nguyen et al. 2022; Park et al. 2018). In brief, QPI measures the change in the phase component of light, providing intrinsic optical imaging contrast. The phase change can be used to determine both the refractive index (RI) and thickness of a sample, allowing identification of subcellular structures with known RI, such as mitochondria and nuclei, thus providing greater specificity. Additionally, the RI and volume measurements can be used to estimate the dry mass of the sample (Barer 1952; Schürmann et al. 2016). In the past, QPI has been limited to the study of single cells since multiple scattering leads to reduced contrast and image quality in thick samples. Recently, the use of the phase-gradient contrast technique (Ford et al. 2012) enabled the reconstruction of quantitative phase information in thick samples, as demonstrated in dissected whole mouse brains, and human liver organoids (Ledwig and Robles 2019; Filan et al. 2024). Notably, tracking of single cells flowing through blood vessels in free-swimming zebrafish larvae exemplified QPI’s high-speed imaging capability (Kandel et al. 2019).

While complex imaging modalities often require in-house instrumentation, this challenge is mitigated by the availability of commercialised QPI systems. This had proved to be invaluable for monitoring preimplantation mouse development, revealing features used for differentiating embryos with varying developmental potentials (Lee et al. 2024b). Applied to intestinal organoids, it has successfully identified different cell types based on morphometric differences used to select viable organoids (Lee et al. 2023). In studying ovarian folliculogenesis ex vivo, QPI offers a unique approach to track GC rearrangement, proliferation and deaths (Fig. 2I). Since the lumen has a distinctly lower RI than that of cellular structures, QPI also holds promise in unravelling the precise dynamics of lumniongenesis. Direct quantification of luminal RI will also potentially inform changes in lumen composition during antral follicle development. Currently, intravital QPI imaging remains challenging due to severe light scattering in optically inhomogeneous biological tissues. However, scattering reduction techniques have successfully retrieved signals from circulating blood cells in the mesentery of live mice (Kim et al. 2016), highlighting the potential for future advancements in imaging dynamic processes in vivo.

Ideally, applying OCT to in vivo contexts will reveal true tissue dynamics in their native state, but it comes with limitations in spatial resolution and setup complexity. Alternatively, multiphoton imaging provides higher spatial detail but is constrained by reduced temporal resolution due to phototoxicity. We advocate the use of OCM and QPI to study ovarian folliculogenesis in ex vivo cultures, which should offer an optimal spatiotemporal resolution that captures the essential features of ovarian dynamics.

A general drawback of label-free imaging techniques discussed above lies in their limited cellular and molecular specificity. Nevertheless, these methods can be combined with fluorescence-based modalities (Mowla et al. 2024; Bevilacqua et al. 2023; Lee et al. 2023; Nguyen et al. 2017). Additionally, the adoption of virtual staining techniques enabled the prediction of both dye and fluorescence signals in label-free images (Fig. 2A) (Winetraub et al. 2024; Park et al. 2023a, b). This integration with the artificial intelligence (AI) approach enhances the specificity and versatility of label-free imaging, opening avenues for comprehensive cellular and molecular characterisation, a topic we will delve into in the following section.

## Machine learning approaches to quantify tissue properties from large datasets

Quantitative studies of tissue dynamics in large systems can generate enormous amounts of data, particularly if the dynamics are fast. In this section, we describe several machine-learning approaches used to investigate biological processes and highlight their potential use in studying late-stage follicle development.

### What is machine learning?

Machine learning comprises a set of algorithms in which a model is trained to complete repetitive sets of tasks (Hallou et al. 2021; Jones et al. 2017; Howard and Gugger 2020). Deep learning is one type of these algorithms, which are vast “artificial neural” networks with the ability to learn complex patterns from the data and solve problems which require extensive manual work (Howard and Gugger 2020). Deep learning has been particularly successful and is increasingly being used in biological and medical imaging (Culley et al. 2023; Hallou et al. 2021; Singh et al. 2020). The major advantage of using these algorithms is they often outperform other automation approaches and that once trained, the model can be applied to vast datasets with minimal or no human input. One method of training the models is by supplying a dataset of labelled examples where a human expert first completes the classification or segmentation task by hand, followed by training the machine learning model to perform a similar task on new data (Howard and Gugger 2020).

### Segmenting and quantifying 3D data

One of the most common applications of deep learning is cell segmentation, which has been extensively applied to tissue morphogenesis, wound healing and tumour segmentation (Turley et al. 2024a, b; Chattopadhyay and Maitra 2022; Işin et al. 2016; McDole et al. 2018; Wang et al. 2022; Mitchell and Cislo 2023; Ichbiah et al. 2023; Boylan et al. 2024). Recently, 3D segmentation models have been used to investigate cell layers surrounding a lumen in MDCK cells (Andrés-San Román et al. 2023) and early stages of sea star embryo development (Barone et al. 2024). Following segmentation, other morphometric information such as cell volume, elongation and packing ratios can be quantified readily. The differences in tissue packing and rates of cell rearrangements can indicate changes to tissue stiffness and rheology, respectively (Lou et al. 2023; Tetley et al. 2019; Stroka and Aranda-Espinoza 2011). Without automated deep learning models, segmentation studies on this vast scale would not be feasible. Such techniques will be highly relevant to quantifying intraofollicular dynamics, where the precise segmentation of oocyte and GC size, shape and movements may lead to new insights on tissue phase transitions during folliculogenesis (see the “Potential phase transitions during antral folicle development” section).

### Virtual stain of label-free data

Label-free imaging techniques, as discussed in the “Quantitative phase imaging” section, can also benefit from deep learning methods. Here, deep learning models can be used to virtually stain or segment regions of the cells such as the nucleus (Christiansen et al. 2018; Jo et al. 2021; Park et al. 2023a). This has recently been demonstrated on images of cells acquired through combined quantitative phase imaging and fluorescence microscopy (Jo et al. 2021). Using fluorescent microscopy images as the ground truth, deep learning models were trained to segment various cytoskeleton components and organelles from the quantitative phase images. This model was highly accurate and even capable of labelling other cell lines which it was not trained on Jo et al. (2021). Importantly, this implies that the model, once trained, can be extended to label cells during live imaging, where the use of dyes may be cytotoxic, or in non-transgenic organisms such as human samples. Hence, we propose that quantitative phase imaging, combined with deep learning, will provide a unique and timely approach to study the dynamics of ovarian folliculogenesis, particularly when the interstitial fluids cannot be labelled with fluorescent tags readily.

### Deep learning models to detect dynamic behaviors and forces

Beyond segmenting and classifying regions of a tissue, deep learning methods can be used to detect dynamic behaviour such as cell divisions and extrusions (McDole et al. 2018; Turley et al. 2024b, a; Villars et al. 2023). The dynamics of cell divisions with daughter nuclei undergoing splitting and cytokinesis are visually distinct from the relatively stationary non-dividing cells. Therefore detection of cell divisions using deep learning models has been highly successful (McDole et al. 2018; Turley et al. 2024b, a; Villars et al. 2023). Combined with additional fluorescent markers labelling cell membrane, the detection of cell division remains robust even in noisy environments. This has been demonstrated in the case of wound healing in *Drosophila* (Turley et al. 2024b, a), where the presence of cell debris and immune cells did not pose a challenge for analysing cell division dynamics through deep learning. So far, deep learning analysis has been larged confined to 2D tissues. The extension of such approach to 3D tissue dynamics, such as ovarian folliculogenesis, will be necessary given the huge datasets and natural biological variation that can only be overcome with machine learning.

AI algorithms have also been used to infer cellular forces from microscopy data (Schmitt et al. 2023). Here, traction force microscopy was used as the ground truth to quantify forces generated by single cells. Based on the confocal images, the information provided by the distribution of zyxin was sufficient to train a deep-learning model to determine the traction forces. Remarkably, even though the model has not been trained on cells treated with cytoskeletal perturbations, it was able to predict the traction stress field in these conditions which matches the experimental results (Schmitt et al. 2023, 2024). Using physics-informed neural networks, these work also demonstrate the unique possibility of constructing a physical model with interpretable physics and parameters (Schmitt et al. 2024; Karniadakis et al. 2021; Colen et al. 2021). Such approach has recently been applied to *Drosophila* embryogenesis (Lefebvre et al. 2024), where the active stress generated by the myosin distribution is sufficient to train the neural networks to predict the tissue flow pattern driving germ-band extension. Importantly, the model can also be trained to “construct” the minimal sets of equations describing the dynamics of the myosin field (Lefebvre et al. 2024), thereby unravelling previously hidden physics underlying such processes. In ovarian follicle development, such a data-driven biophysical modelling approach may provide an exciting alternative approach to understand the underlying physical principles governing robust folliculogenesis.

### Unbiased pattern detection in tissues without supervision

In addition to quantifying cellular features, deep learning methods could also inform how these properties change in time and space. Here, a certain kind of deep learning model based on unsupervised learning techniques (Zinchenko et al. 2023; Lu et al. 2019; Lafarge et al. 2019) provides an unbiased approach to distinguish the distinct morphological features from different cell types. This was recently applied to electron microscopy data from a marine annelid (*P. dumerilii*), where the model extracted features of both cell shape and texture using dimensionality reduction techniques (Zinchenko et al. 2023). In this “MorphoFeatures” space, clear clusters of cell types can be extracted, which demonstrates that the model has learned to distinguish individual cell types without supervision. In addition, the features extracted by the deep learning model can further reveal the unique cellular modules defining the different cell types (Zinchenko et al. 2023). In the study, different cell types were used, but similar techniques may be applied to track the different stages of development for a given cell type. In future, it will be exciting to apply such models to identify changes in GC identity during ovulation or to compare GC or oocyte features in follicles from young, old and diseased ovaries in an unbiased manner.

## Theoretical approaches to study the physics of antrum formation and ovulation

In the past decades, various theoretical approaches have been developed to study tissue morphogenesis, wound healing and cancer progression (Tse et al. 2012; Jacques et al. 2023; Etournay et al. 2015; Salbreux et al. 2009; Tetley et al. 2019; Turley et al. 2022). These approaches modelled biological systems as active soft matter, where individual cells are able to convert chemical energy to execute various biological functions (Lou 2023; Marchetti et al. 2013; Fuji et al. 2022). Here, we review recent in silico work and biophysical approaches to model and measure tissue hydraulics, and propose new theoretical frameworks to investigate antrum formation and ovulation in late-stage folliculogenesis.

### Computational models of luminogenesis

While the cellular dynamics in tissues have been modelled extensively, theoretical work on modelling lumen remains rather limited. Recently, a number of computational approaches have been developed to study lumen growth in development and organoids. One such approach is the phase field model, which can model cellular and lumen dynamics (Akiyama et al. 2018; Nonomura 2012; Fuji et al. 2022; Tanida et al. 2024). Phase field model has recently been applied to study the formation of MDCK cysts, pancreatic spheres and epiblasts, revealing a generic rule of the two-phase process of luminogenesis (Lu et al. 2024) characterised by lumen nucleation mediated by actin polymerization (Vasquez et al. 2021; Mukenhirn et al. 2023; Indana et al. 2024), and later expansion via osmotic gradient (Indana et al. 2024). The phase field model also reveals the interplay between cell proliferation and lumen pressure in changing organoid topology, as in the case of pancreatic spheres or branched networks (Lee et al. 2024a). Another popular approach in modelling luminogenesis is the Cellular Potts model (Graner and Glazier 1992; Hirashima et al. 2017). This technique has been applied to study organ cystogenesis (Belmonte et al. 2016; Engelberg et al. 2011) and development (Mombach et al. 2001). Recent work has extended the Cellular Potts model to include non-conservative forces arising from active cellular fluctuations (Belousov et al. 2024), which can be a novel approach to model non-equilibrium aspects of luminogenesis.

### Potential phase transitions during antral folicle development

A key application of physics to the study of biological systems is the modelling of tissues as fluid- and solid-like materials, as reviewed in Lenne and Trivedi (2022). These theoretical models have predicted key cellular parameters that describe the mechanical states of tissues, and the critical points where phase transitions may occur. These parameters include cell elongation, active fluctuations and changes in extracellular space between the cells (Mongera et al. 2018; Petridou et al. 2021, 2019). An example is blastoderm spreading in early zebrafish development, where the center of the dome fluidises while the margin of the dome remains solid-like (Petridou et al. 2019). As the volume fraction of interstitial fluid increases and the cellular network breaks down, the marginal tissue becomes more fluid-like, leading to tissue flow. Here, we speculate that similar physics may apply to secondary follicles undergoing luminogenesis, where decreased GC packing may lead to increased tissue fluidisation (Biswas et al. 2022; Telfer et al. 2023). Tissue fluidization brings about another type of phase transition during antrum formation, which is the mixing-demixing transition in a fluid mixture. Here, the lumen and GCs could be considered effectively as a binary mixture of fluids, and luminogenesis may be modelled as liquid-liquid phase separation. While previous Cellular Potts model or phase field models have simulated lumen dynamics in various contexts, it is often assumed *a priori* that the systems exist far from criticality. Here, we hypothesise that the antrum formation in ovarian follicles may involve a phase transition from critical to supercritical states as the lumen undergoes fusion and maturation.

To test these models, it is essential to develop experimental approaches to measure and perturb tissue mechanics in follicles. Recently, 3D force sensors like polyacrylamide microbeads have been developed to measure mechanical stress distribution within cancer cell spheroids, revealing that an increased tissue pressure toward the inner core leads to inhibition of cell proliferation (Dolega et al. 2017; Taubenberger et al. 2019). Interestingly, tissue pressure has recently been shown to impact GC proliferation and follicle growth in the secondary follicle stage (Biswas et al. 2024, 2022), raising the intriguing possibility that intrafollicular pressure may impact subsequent events such as luminogenesis.

### Biophysical control of ovulation

While it is well known that ovulation is triggered by hormonal signalling, the dynamics and mechanics of this process remain poorly characterised. Early studies have proposed that various mechanical factors, such as follicle volume increase, degradation of follicle wall and a build-up of hydrostatic pressure, may all be involved in ovulation (Matsuzaki 2021; Rondell 1970). However, these hypotheses remain to be tested. Here, a first characterisation of tissue mechanics during ovulation, such as changes in hydrostatic or osmotic pressure of the antrum by micropressure probes or pressure sensors may be instructive (Matousek et al. 2001; Espey and Lipner 1963; Bronson et al. 1979; Vian et al. 2023; Chan et al. 2021). Measurement of follicle wall tension or stiffness by micropipette aspiration or atomic force microscopy could also provide physical parameters to support model construction. Recently, new imaging-based tools to probe tissue stiffness have emerged. For example, optical coherence elastography (OCE), which is an extension of OCM (see the “Optical coherence tomography” section), relies on measuring local strain and stress to derive elasticity upon compressive load application (Li et al. 2021). OCE has been applied to study cancer cell metastasis, where the peripheral cells in breast cancer spheroids were shown to soften the surrounding ECM leading to invasive migration (Mowla et al. 2023). OCE has also revealed changes in mechanical properties of corpora lutea and follicles during ovarian ageing (Hepburn et al. 2024). Regardless, OCE measurements require the application of a load on the sample, which can result in changes of the sample itself. An alternative is the use of contact-free Brillouin microscopy, which measures the longitudinal modulus of tissue with submicron resolution (Prevedel et al. 2019). Recent work using Brillouin microscopy has revealed the emergence of distinct mechanical compartments within follicles during development (Fig. 2G) (Chan et al. 2021). With the recent advancement in Brillouin microscopy (Bevilacqua et al. 2023), rapid 3D live mapping of tissue material properties is now possible, paving the way for future application of such technique in studying basement membrane remodelling during ovulation.

An interesting biophysical aspect of ovulation is inspired by the physics of liquid crystals (Marchetti et al. 2013; Salbreux et al. 2009; Olenik et al. 2023; Colen et al. 2021; Popović et al. 2017). These frameworks are relevant when elongated cells align with each other and create long-range nematic order in tissues, as observed in fibroblasts, myoblasts and epithelial cells (Saw et al. 2017; Duclos et al. 2016; Sonam et al. 2022). In cases where apical-basal polarisation occurs, such polar order can further form topological defects in 3D, as observed during luminogenesis of the inner cell mass at the mouse pre-implantation stage (Guruciaga et al. 2024; Ichikawa et al. 2022). During *Hydra* regeneration, long, super-cellular actin bundles can form nematic order (Ravichandran et al. 2024; Maroudas-Sacks et al. 2021). The “closed” topology of this tissue implies that topological defects must be present. Interestingly, such topological defects have implications in *Hydra* development, where breaches only occur at these defects to relieve tissue pressure (Ravichandran et al. 2024), supporting the notion that topological defects can act as mechanical organisers during morphogenesis (Ravichandran et al. 2024). We propose that similar physics may apply to ovulation, where the spindle-like TCs may arrange into a nematic state and exhibit potential topological defects that facilitate follicle rupture during ovulation.

## Conclusions and perspectives

In this review, we introduced the latest developments in microscopy, machine learning approach and theoretical models that may advance our understanding in late-stage mammalian folliculogenesis. We foresee that such techniques may lead to immediate new findings when applied to follicles grown ex vivo, but studying follicle development in vivo may pose additional challenges due to sample size and multiscale complexity. To move beyond classical histological and descriptive studies, and to have a more comprehensive and quantitative understanding of mammalian folliculogenesis, a multidisciplinary approach combining biophotonics, biophysics and machine learning is essential (Fig. 2).

Individually, the methods discussed above can provide insights but when integrated together they become more powerful. Advanced microscopy informs microscopic interactions between cells. This qualitative information can be transformed into quantified data using deep learning models, done accurately and efficiently on a large scale. Biophysical tools can be used to measure macroscopic properties of the tissue and theory can be deployed to understand how the microscopic interactions can produce macroscopic effects. Perturbations of the models can then be tested using the same quantitative tools to validate the theory. We envision this as an iterative process with advanced imaging experiments and analyses informing biophysical modelling, followed by a theoretical approach guiding further experiments and generating new hypotheses for testing. The use of novel biophotonic tools to directly infer tissue mechanics (e.g., Brillouin microscopy) also facilitates construction of biophysical models. Such an integrative framework to study 3D tissue dynamics will also be useful in the study of organoids and disease models (Beghin et al. 2022).

In addition to deepening our knowledge of female reproductive biology, a quantitative understanding of tissue hydraulics in late-stage folliculogenesis also has profound clinical implications. For example, it will be interesting to compare antrum formation and ovulation dynamics in the young and old follicles for possible biomechanical origin of infertility. Similarly, extending these approaches to ovarian disease models, such as PCOS ovaries, will shed light on the potential misregulation of tissue hydraulics in these systems.

## Data Availability

Not applicable
